# Regulation of Sugar Metabolism During Fermentation of Brewers' Spent Grain by *Leuconostoc pseudomesenteroides* DSM20193

**DOI:** 10.1111/1751-7915.70116

**Published:** 2025-04-21

**Authors:** Koirala Prabin, Maina Ndegwa, Mojzita Dominik, Coda Rossana

**Affiliations:** ^1^ Helsingin Yliopisto Helsinki Finland; ^2^ Teknologian Tutkimuskeskus VTT Oy Espoo Finland; ^3^ Sustainability Science (HELSUS), Faculty of Agriculture and Forestry University of Helsinki Helsinki Finland

**Keywords:** Brewers' spent grain, dextran, fermentation, lactic acid bacteria, Leuconostoc, sugar metabolism, transcriptome analysis

## Abstract

Re‐utilising brewers' spent grain (BSG) through LAB fermentation can enable its broad use in the food industry, enhancing its nutritional and functional properties and offering a clear example of a sustainable approach in the valorisation of food side streams. Despite extensive research on LAB fermentation, the regulation of metabolism during the growth in complex food‐industry‐relevant environments remains unclear. This study investigates the metabolic processes in *Leuconostoc pseudomesenteroides* DSM20193 during 24 h fermentation of BSG with and without 4% sucrose (w/w) supplementation, allowing in situ dextran synthesis. Besides dextran synthesis, the presence of sucrose led to faster acidification, especially due to the increased formation of acetic acid. Furthermore, differences in the utilisation of sucrose, fructose, glucose, and maltose and the formation of diverse oligosaccharides were observed. Transcriptome analysis comparing expression profiles during 0 h and 16 h growth in BSG with sucrose revealed differences in the expression of genes involved in carbohydrate utilisation pathways, including higher activity of sucrose and maltose metabolism and lower activity of metabolism related to alternative carbon sources. Transcription analysis of selected relevant genes in a time‐course comparison between BSG with and without sucrose provided more detailed indications of responses of the metabolic network in this complex environment. This analysis provided a deeper understanding of the dynamic regulatory mechanism that drives sugar metabolism and dextran synthesis and how the presence of sucrose can alter the metabolic flux towards different fermentation products.

## Graphical abstract

This study reveals the regulatory mechanism behind sugar metabolism and dextran synthesis by *Leuconostoc pseudomesenteroides* DSM20193 during the fermentation of a complex substrate like brewers spent grain. Understanding metabolic pathways and genetic adaptation of lactic acid bacteria in food environments can help develop fermented products with desired properties.

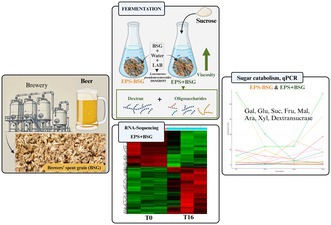

## 1 Introduction/Background

Lactic acid bacteria (LAB) are gram‐positive bacteria commonly used in the food industry to produce fermented foods and lactic acid (Costa et al. 2020; Sheeladevi and Ramanathan 2012). Their capacity to utilise different carbohydrates and wide metabolic adaptability has put them at the forefront of comprehensive research on metabolic performance (Buron‐Moles et al. 2019; Liu et al. 2019). The LAB fermentation process is a technology able to add value to food production side streams or by‐products, enhancing their nutritional and functional properties (Sun et al. 2022; Verni et al. 2019). The exploration of alternative substrates for LAB fermentation underscores the need to broaden the research beyond traditional food media to fully employ the potential of LAB in utilising diverse agro‐food residues. One such important substrate that has gained increasing attention in recent years is brewers' spent grain (BSG), a fibre‐rich by‐product of the brewing industry with a limited amount of readily available sugars (Acin‐Albiac et al. 2022; Eliopoulos et al. 2022; Fărcaș et al. 2021). LAB fermentation of BSG has been explored to improve its technological properties, i.e., rheological properties, extend the shelf life, and increase its bioactivity (Ktenioudaki et al. 2015; Magabane 2017; Neylon et al. 2021; Verni et al. 2019).

For instance, one approach to achieve texture modification of BSG is via the synthesis of dextran during LAB fermentation (Koirala et al. 2021, 2022). Dextran is a complex, branched polysaccharide composed of glucose monomers (Kothari et al. 2015; Robyt et al. 2008) with diverse applications in the food industry (Kothari et al. 2015; Naessens et al. 2005; Wang, Maina, et al. 2021; Wang, Wu, et al. 2021). As a hydrocolloid, dextran has emulsification properties, water retention ability, and the capacity to increase viscosity, consequently altering the texture of raw materials. Additionally, dextran has shown a positive influence on human gut microbiota, enhancing the production of beneficial short‐chain fatty acids (SCFAs) (Al‐Khafaji et al. 2020; Koirala et al. 2022; Kumar et al. 2020; Makki et al. 2018). Apart from the different intrinsic dextran production capacities of diverse LAB species, the fermentation conditions play an important role in dextran formation (Daba et al. 2021; More et al. 2014). The composition of the fermented substrate, and in particular, the presence of different carbon sources (sugars), strongly influences the metabolic activity of LAB. Carbon catabolite repression (CCR) and other regulatory mechanisms allow bacteria to adapt to the changing conditions in complex environments—preferentially metabolising specific carbon sources at specific phases of fermentation, with profound implications for growth and product formation (Brückner and Titgemeyer 2002; Mahr et al. 2000; Rojo and Alejandro Dinamarca 2004; Ruiz‐Villafán et al. 2022).

During dextran synthesis, dextransucrase catalyses the breakdown of sucrose to glucose and fructose, where glucose is utilised for the concomitant dextran synthesis, while free fructose accumulates (Naessens et al. 2005; Robyt et al. 2008). As an example of metabolic regulation during this process, upregulation of the expression of the fructokinase‐encoding gene was observed during dextran production in the presence of sucrose (Helanto et al. 2006; Li et al. 2021). LAB show broad adaptive responses to altering sugar availability by upregulating diverse enzymes needed for complex sugar metabolism. For instance, an increase in the production of 6‐phospho‐beta‐glucosidase (6pbg) and fructose‐6‐phosphate phosphoketolase (F6PPK) was observed in conditions when readily available sugars such as glucose became limiting, and the metabolism switched to utilisation of sugars such as cellobiose, xylose, or arabinose derived from complex lignocellulosic biomass. These metabolic regulations are essential during the hydrolysis of complex carbohydrate substrates, allowing the balanced and timely use of available carbon sources (Acin‐Albiac et al. 2021; Zeidan et al. 2017; Zhang, Perez‐Gavilan, et al. 2023).

Different species within the genus *Leuconostoc* display varied sugar utilisation profiles; for example, *Leuconostoc mesenteroides* preferentially metabolises sucrose, whereas *Leuconostoc lactis* primarily utilises glucose (Besrour‐Aouam et al. 2021; Gänzle 2015; Huang et al. 1994). These preferences are attributed to species‐ and strain‐specific differences in genetic makeup, but also to differential gene and enzyme activity regulations, which are modulated in response to the environment (Doran‐Peterson et al. 2008; Kim et al. 2010; Zaunmüller et al. 2006). Distinct sugar consumption profiles and different metabolic responses during BSG fermentation with different LAB species, especially *Leuconostoc pseudomesenteroides* and *Weissella confusa* , were demonstrated earlier (Acin‐Albiac et al. 2021; Koirala et al. 2021; Xu et al. 2018). The availability of sugars in BSG significantly influenced the fermentation outcome, underlining the adaptability of LAB in diverse environments (De Vos 1996; Gänzle et al. 2007; Luesink et al. 1998; Zeidan et al. 2017). Most of the studies about the regulation of sugar catabolism by LAB have been performed during growth in MRS, a standard growth medium rich in peptones and sugars optimised for the growth of LAB (De Man et al. 1960; Ummadi and Curic‐Bawden 2010; Yeboah and Krastanov 2023). However, reliance on MRS medium limits the understanding of the true capabilities of LAB during the fermentation of complex agro‐food‐derived substrates (Charalampopoulos et al. 2002; Hayek and Ibrahim 2013; Paramithiotis et al. 2007); only a few studies have used substrates simulating food or modified MRS (Acin‐Albiac et al. 2022; Adebayo‐Tayo and Onilude 2010; Hayek et al. 2019).

A recent study investigated how LAB metabolise complex BSG‐derived substrates and adapt to the environment using a phenomic micro‐array approach (Acin‐Albiac et al. 2021). In those conditions, *L. pseudomesneteroides* DSM20193 displayed a distinctive ability to metabolise d‐xylose, sucrose, and cellobiose, observed through overexpression of xylA, INV, and pbg‐like genes, while other species (*Lactiplantibacillus plantarum*) preferred lactose, galactose, or l‐arabinose and d‐gluconic acid depending on the strain (Acin‐Albiac et al. 2021).

In this study, we investigated the fermentative performance and regulation of the sugar catabolism genes in *L. pseudomesenteroides* DSM20193 during BSG fermentation under conditions conducive to dextran synthesis. Growth and acidification kinetics, viscosity, sugar‐related metabolism, and dextran and oligosaccharide synthesis were assessed. Whole genome transcriptome (RNAseq) as well as quantitative PCR‐based transcription analysis were employed to monitor the regulation of dextransucrase and the key sugar catabolism genes and to elucidate the bacterial adaptive responses to varying BSG sugar composition.

## 2 Materials and Methods

### 2.1 Media and Cultivation Conditions

*Leuconostoc pseudomesenteroides* DSM20193 was obtained from the Leibniz Institute DSMZ (Braunschweig, Germany) and was routinely cultivated in MRS broth (NEOGEN, UK) at 30°C for 24 h.

The BSG used for this study was provided by Dugges Bryggeri (Dugges Bryggeri AB, Landvetter, Sweden). BSG was collected immediately after the brewing process and stored at −20°C before use. Before fermentation, BSG was thawed at room temperature and subjected to wet milling (Microcut MC15, de man K04 blade, Stephan Machinery GmbH, Hameln, Germany). Milling was performed twice, resulting in a fine BSG paste. Milled BSG had 80.5% moisture and 20.9% dry matter (AACC method 44‐15.02). Dried BSG had a composition of 9.9% fat, 23.4% protein, and 47.9% total dietary fibre.

MRS (De Man et al. 1960) broth (NEOGEN, UK) was used for routine starter cultivation and preparing inoculum for BSG fermentation. *L. pseudomesenteroides* DSM20193 was cultivated in MRS broth for 24 h. Bacterial cells were centrifuged (10,000 rpm for 10 min at RT) and washed once with 1× PBS (pH 7.4). Cell pellets were re‐suspended in 500 μL Milli‐Q water and used to inoculate the BSG‐water mixture, targeting an initial cell density of ca. 6.0 Log CFU/g. For fermentation, 50 g of BSG and 80 mL of water were mixed in a beaker. Cells were inoculated in the BSG and water mixture (defined as EPS−); to enable dextran synthesis, 10% w/w of BSG was substituted with sucrose (defined as EPS+) in consistency with our previous study (Koirala et al. 2021). Fermentations were performed at 25°C for up to 24 h. Control samples with and without sucrose, but without starter inoculum, were prepared as described above and incubated at 25°C for 24 h.

During fermentation, aliquots of samples were collected for bacterial enumeration, pH, organic acid analysis, and sugar analyses at selected time points. Kinetics of bacterial growth and acidification were monitored during 24 h of BSG fermentation at the following time points: T0, T8, T10, T16 and T24 (in hours). Total microbial RNA was extracted from fresh aliquots of fermented BSG for the whole genome transcriptome (RNA sequencing) and targeted transcription (quantitative PCR analysis—qPCR) analyses.

### 2.2 Bacterial Enumeration, pH, Total Titratable Acidity (TTA), and Organic Acid Analysis

Microbial enumeration was performed by homogenising 10 g of BSG mixture with 90 mL of sterile 0.9% w/v NaCl solution using a stomacher (Colworth, UK) and preparing serially diluted suspensions, which were plated on specific agar plates as described previously (Koirala et al. 2021). The total mesophilic bacteria, presumptive LAB, *Bacillus cereus* , *Enterobacteriaceae*, yeasts, and moulds were monitored before and after 24 h of fermentation. Presumptive LAB was also determined from homogenised BSG control samples without starter inoculum.

The pH of fermented BSG was measured with an online pH meter (Knick, Germany). TTA was evaluated using an automatic pH titrator (Easy Plus, Mettler Toledo, Columbus, OH, USA); the sample was prepared with the modified AACC method 02‐31.01, as described previously by Koirala et al. (2021). TTA was quantified by measuring the volume of 0.1 N NaOH required to reach a pH of 8.5.

The amount of lactic and acetic acid was determined using a high‐performance liquid chromatography (HPLC) system, as described previously (Xu et al. 2017). For sample preparation, 4 g of fermented and homogenised samples were mixed with Milli‐Q water, vortexed for a few minutes, and centrifuged at 10,000 rpm for 10 min. Supernatants were filtered through a 0.45 μm filter (Pall, USA) prior to the HPLC analysis.

### 2.3 Viscosity, Dextran, Saccharides, and Mannitol Analysis

The viscosity of the fermented BSG samples was measured at different time points (T0, T8, T10, T16, and T24) of the fermentation. The viscosity was analysed with the RheolabQC Rheometer (Anton Paar, Austria) by transferring homogenised samples into the measuring cup C‐CC27/QC‐LTD (Anton Paar, GmbH) and using the CC27 probe (Anton Paar, diameter: 26.665 mm, length: 39.977 mm and concentricity: ±4 μm) at a constant temperature of 22°C. After transferring samples into the measuring cup, the samples were rested for 5 min at room temperature before measurement. Viscosity flow curves were recorded starting at a shear rate of 3 to 100 S^−1^ and back (33 points each direction). At 100 S^−1^, ten measurement points were recorded with 5 s intervals between each measurement. The average viscosity (mPa.S) at a constant shear rate of 100 S^−1^ was used to compare the viscosity of the samples.

Dextran was quantified from fermented BSG at selected time points (T0, T8, T10, T16, and T24) by an enzyme‐assisted method as previously described (Katina et al. 2009) using a mixture of two enzymes, dextranase (Sigma‐Aldrich, Denmark) and transglucosidase (Megazyme, Ireland). Glucose (Merck, Germany) was used as the standard, and 2‐deoxy‐d‐galactose (Sigma‐Aldrich, UK) was used as the internal standard for quantification.

Samples for oligosaccharides, sugars (mono‐ and disaccharides) and mannitol were prepared by homogenising 100 mg of freeze‐dried fermented BSG in 5 mL milli‐Q water in a 15 mL falcon tube. The tube was incubated in a boiling water bath for 10 min and then centrifuged at full speed (at +4°C) for 10 min. The supernatant was filtered through Amicon Ultra Centrifugal Filters 0.5 mL 10 K (Merck Millipore Ltd., Ireland). Finally, the filtrate was transferred into the vials, and an internal standard (xylotriose, Koirala et al. 2022) was added and used for the quantification. The oligosaccharides (or maltosylisomaltooligosaccharides, MIMO) profile was analysed as reported previously (Koirala et al. 2022) using panose (pan) and maltooligosaccharides (M3: maltotriose, M4: maltotetraose, M5: maltopentaose, M6: maltohexaose, and M7: maltoheptaose) as standards.

Free mono‐ and disaccharides were quantified from fermented BSG using a high‐performance anion exchange chromatography with pulsed amperometric detection (HPAEC‐PAD) system. The carbohydrates were separated using a CarboPac PA1 column (250 × 4 mm i.d. Dionex, Sunnyvale, CA) and detected with a Waters 2465 pulsed amperometric detector (Waters, USA). The gradient elution method was applied to separate different carbohydrates using mobile phase A (200 mM NaOH) and mobile phase B (MQ water) at a flow rate of 1 mL/min and at a 10°C column temperature. The applied gradient run was 60 min, starting with 1% of mobile phase A and 99% of B for 4 min, 30% A and 70% B until 30 min, 100% A until 38 min, remaining at 100% A until 48 min, 1% A and 99% B until 50 min, and stabilising for the final 10 min at 1% A and 99% B. Galactose, glucose, sucrose, fructose, maltose (Merck, Germany), arabinose (Sigma‐Aldrich, UK), and xylose were used as standards, and 2‐deoxy‐d‐galactose (Sigma‐Aldrich, UK) was used as an internal standard. Mannitol was quantified from fermented BSG samples as described previously (Xu et al. 2017).

### 2.4 RNA Extraction and cDNA Synthesis

Total bacterial RNA extraction from fermented BSG was done at selected time points T10, T16, and T24, where relative changes in viscosity were observed (Table 1). As the representative of T0, RNA was extracted from the MRS broth culture of the starter grown for 24 h and used for inoculation. For RNA extraction from fermented BSG, 60 g of fermented BSG were collected and mixed with an equal amount of RNA later Stabilisation Solution (Invitrogen, Lithuania), homogenised, and left overnight at 4°C. The sample was mixed with 60 mL sterile 1× PBS and vacuum filtered through two layers of Miracloth (Millipore, Merck) to remove coarse particles of BSG. Fifty ml of filtrate was centrifuged at 500 × *g* for 1 min at 4°C to remove fine particles of BSG. The cells were finally obtained as pellets after centrifugation at 4000 × *g* for 5 min at 4°C, re‐suspended in 1 mL of buffer RLT (RNeasy Mini Kit, Qiagen, Germany), and transferred into 2 mL screw‐cap tubes containing ca. 600 μL of acid‐washed 425–600 μm glass beads (G8772‐500G, Sigma‐Aldrich). Mechanical disruption of cells was performed in FastPrep‐24 (MP Bio‐chemicals) at max speed (6.5 m/s) for 30 s, and the lysates were centrifuged at maximum speed for 3 min at 4°C. Extraction and purification of total RNA from the clarified supernatants were performed with the RNeasy Mini Kit according to the manufacturer's instructions. Post‐extraction RNA precipitation was done using 3 M Sodium Acetate (pH 5.2–5.5) and Propan‐2‐ol as reported previously (Hughes 2019). RNA concentration and quality were determined using a NanoDrop 1000 Spectrophotometer (Thermo Scientific).

**TABLE 1:** Changes in pH, total titratable acidity (TTA), organic acids (lactic and acetic), bacterial count, and viscosity (millipascal per second) observed at different time points (T0, T8, T10, T16 and T24) of fermentation of BSG in conditions with (EPS + BSG) and without (EPS‐BSG) dextran synthesis.

Time points	pH	TTA	Organic acids (mg/100 g BSG, d.w.)		Bacterial count (Log CFU/g)		Viscosity (mPa.S)
			Lactic acid	Acetic acid	pLAB	TMB	
**EPS‐BSG**							
T0	6.0 ± 0.1^a^	0.8 ± 0.03^a^	nd		6.8 ± 0.1^a^	6.8 ± 0.02^a^	49.5 ± 0.7^a^
T8	5.6 ± 0.04^b^	1.4 ± 0.1^b^	422.2 ± 31.9^a^	115.4 ± 4.2^a^	8.2 ± 0.1^b^	8.7 ± 0.6^b^	35.6 ± 0.7^b^
T10	5.3 ± 0.04^b^	1.4 ± 0.03^b^	406.5 ± 20.3^a^	123.7 ± 3.9^b^	8.7 ± 0.2^c^	9.1 ± 0.1^c^	35.0 ± 0.3^b^
T16	4.6 ± 0.1^c^	2.4 ± 0.04^c^	1276.1 ± 28.5^b^	106.5 ± 1.4^c^	9.2 ± 0.1^d^	9.8 ± 0.2^c^	46.7 ± 1.6^a^
T24	4.3 ± 0.1^d^	3.6 ± 0.2^d^	1461.8 ± 30.3^c^	135.4 ± 0.6^d^	9.2 ± 0.2^d^	9.2 ± 0.2^d^	38.3 ± 1.5^b^
**EPS + BSG**							
T0	6.0 ± 0.2^a^	0.7 ± 0.1^a^	nd		6.7 ± 0.2^a^	6.6 ± 0.02^a^	44.3 ± 1.0^a^
T8	5.2 ± 0.1^b^	1.8 ± 0.1^b^	360.3 ± 148.5^a^	128.7 ± 35.7^a^	8.5 ± 0.1^b^	8.5 ± 0.2^b^	47.9 ± 1.2^a^
T10	4.9 ± 0.1^b^	2.0 ± 0.1^b^	482.2 ± 21.7^a^	182.9 ± 13.1^a^	8.9 ± 0.1^c^	9.4 ± 0.2^c^	188.8 ± 1.4^b^
T16	4.2 ± 0.1^c^	3.6 ± 0.2^c^	915.1 ± 19.4^b^	423.1 ± 3.7^b^	9.1 ± 0.1^c^	9.8 ± 0.03^d^	454.5 ± 6.8^c^
T24	3.9 ± 0.2^c^	5.5 ± 0.1^d^	1608.5 ± 176.6^c^	761.2 ± 168.2^c^	9.1 ± 0.01^c^	9.4 ± 0.02^c^	831.6 ± 5.7^d^

*Note:* Values in the same column of each fermenting condition (EPS‐BSG and EPS + BSG) with different letters are significantly different (*p* < 0.05).

Abbreviations: nd, not detected; pLAB, presumptive lactic acid bacteria; TMB, total mesophilic bacteria; TTA, expressed as ml of 0.1 N NaOH.

The cDNA (complementary DNA) for use in the qPCR analysis was synthesised from the purified total RNA by the First Strand cDNA Synthesis Kit (Roche) following the manufacturer's instructions, a protocol using random hexamer primers.

### 2.5 Transcriptome Analysis—RNA Sequencing

The transcriptome analysis was performed on the RNA extracted from the MRS broth culture of the starter grown for 24 h and EPS + BSG fermented for 16 h. The total RNA (triplicates) was isolated and shipped to Novogene Co. Ltd. (UK) for the actual analysis. Here is a brief description of the procedures performed by Novogene. For the library construction, ribosomal RNA was removed from the total RNA by ethanol precipitation. The remaining RNA was fragmented, and the double‐strand cDNA was synthesised. The library was then constructed through end repair, A‐tailing, adapter ligation, size selection, USER enzyme digestion, amplification, and purification. The samples coded with an index were sorted into clusters per the manufacturer's instructions. Once the clusters were generated, the library preparations were sequenced on an Illumina platform, creating paired‐end reads. A multi‐step approach was utilised to analyse the data. Fastp was used to process the raw data in FASTQ format, eliminating adapters, poly‐N sequences, and low‐quality reads to obtain clean data. The quality of the clean data was assessed based on Q20, Q30, and GC content to ensure high standards. Clean reads were then aligned to the reference genome ( *L. pseudomesenteroides* KCTC 3652; accession numbers AEOQ00000001 to AEOQ00001160 in the NCBI genome database) using Bowtie2, and novel genes, operons, and transcription start sites (TSS) were identified by applying Rockhopper software. Promoters were predicted by extracting upstream sequences of the TSS. Gene expression levels were quantified, and FPKM values were calculated using the FeatureCounts program. Differential expression analysis was performed using DESeq2 and edgeR, adjusting the false discovery rate and establishing significance thresholds. GO and KEGG enrichment analyses were conducted to identify significantly enriched terms and pathways associated with differentially expressed genes. UTR sequences were predicted using TSS and translation start site information, using tools such as RBSfinder and TransTermHP for Shine‐Dalgarno (SD) and terminator sequence prediction. Additionally, sRNA targets were predicted using IntaRNA, and RNA secondary structures were predicted using RNAfold. Finally, comprehensive mutation analysis was conducted using Picard tools, Samtools, GATK, and SnpEff to detect and annotate variants, including SNPs and INDELs. More details on the library preparation, sequencing, and data analysis are available in Table S1.

### 2.6 Transcription Analysis—Quantitative PCR (qPCR)

To extend the transcriptome analysis findings, a time‐course transcription analysis was performed on the RNA extracted from EPS + BSG and EPS‐BSG fermentations. Ten key gene targets were selected for their relevance to the metabolism of sugars in the BSG cultivations. The genome of *L. pseudomesenteroides* KCTC3652 (accession numbers AEOQ00000001 to AEOQ00001160; NCBI genome database) was used as a source of the sequences of the selected genes and as a template for the qPCR primer design. The primer pairs (Table S1) were designed with the Primer3web software (version 4.1.0). Constitutively expressed gene encoding recombinase protein A (recA) was chosen as an internal control for normalisation and quantification (Marco and Kleerebezem 2008). Quantitative PCR was performed on the Stratagene Mx3000P instrument using KAPA SYBR FAST One‐Step qRT‐PCR Master Mix (KAPABIOSYSTEMS) according to the manufacturer's instructions. Results were analysed by the Stratagene MxPro QPCR Software (Version 4.10); the qPCR conditions are described in Table S3. Fold expression values under each time point of the fermentation (T0, T10, T16 and T24) were determined as relative to the expression of the reference gene, recA (Marco and Kleerebezem 2008). Fold gene expression was calculated using the cycle threshold (Ct) values obtained during the qPCR with formula 2^−DCt^, where DCt is the Ct value of the gene of interest minus the Ct value of the reference (recA) gene (Livak and Schmittgen 2001).

### 2.7 Statistics

The experiments were conducted as three biological replicates and analysed at least twice. qPCR was performed in quadruplicates. The data obtained were analysed using two‐way ANOVA, and the mean comparison was determined using Tukey's test (*p* < 0.05) with the help of SPSS version 25 software. The statistical analysis for the transcriptome analysis (RNA‐seq) was performed at Novogene (Table S1).

## 3 Results

### 3.1 Microbial Growth and Organic Acid Accumulation

Native, wet‐milled brewer's spent grain (BSG) was used as a substrate for the fermentations. First, the indigenous presumptive LAB cell density in the native BSG was determined to be < 3 Log CFU/g. After inoculation with *L. pseudomesenteroides* DSM20193, the density of LAB was ~6.7, and the count gradually raised to ~9.2 Log CFU/g at 24 h of fermentation in both EPS+ (with 4% sucrose addition) and EPS‐ (without sucrose) fermented BSG (Table 1). Total mesophilic bacteria (TMB) count followed a similar pattern in both conditions, reaching maximal counts of ~9.8 Log CFU/g at 16 h of fermentation, followed by a minor decrease at 24 h. *Enterobacteriaceae*, *Bacillus cereus* , yeasts, or moulds were not detected at any stage of the fermentations. The initial pH of both EPS+ and EPS− samples was 6.0, and it dropped to 4.3 and 3.9 after 24 h of fermentation in EPS− and EPS+ fermented BSG, respectively. A gradual pH decrease was observed during the fermentations, with a more progressive drop in the presence of added sucrose (EPS+) (Table 1). The TTA values before fermentation were similar for both conditions (ca. 0.7 mL of 0.1 N NaOH) and reached 3.6 and 5.5 mL of NaOH after 24 h of fermentation in EPS‐ and EPS+ BSG, respectively. TTA values increased gradually in both conditions, with significant increases at 16 and 24 h. Lactic acid was not detected at T0, but it readily accumulated in both conditions, reaching the highest value at 24 h in the fermented EPS+ BSG (1608.5 mg/100 g BSG dry weight, d.w.). However, the production of lactic acid seemed somewhat faster at earlier time points during the EPS‐G fermentation. Similarly, acetic acid was not present at T0, but it was detected at T8 in the EPS‐G samples (115.4 mg/100 g BSG d.w.) and remained relatively stable with a minor increase at T24. In contrast, in the EPS+ fermented samples, acetic acid concentration progressively increased during cultivation, reaching 761.2 mg/100 g BSG at 24 h (Table 1).

### 3.2 Viscosity, Dextran Production, and Sugar Analysis

The viscosity values remained stable throughout the EPS‐BSG fermentation process, showing no significant increase, and ranged between 35.0 and 49.5 mPa.s (Table 1). On the contrary, during the EPS + BSG fermentation, a significant build‐up in viscosity was observed as the fermentation progressed, starting from 44.3 mPa.s at T0 and reaching 831.6 mPa.s at 24 h of the fermentation.

Dextran production was not observed during the fermentation of EPS‐BSG, while it was detected in EPS + BSG after 8 h, reaching 8.5% (d.w.) and corresponding to 50.7% of the maximum theoretical yield of 17.3% based on the amount of added sucrose (34.6%) (Koirala et al. 2021). Theoretical dextran yield is calculated based on the total amount of sucrose added during fermentation, i.e., 34.6%, which gets hydrolysed into 50% glucose and 50% sucrose, with 50% of glucose potentially used for dextran synthesis. The amount of dextran continued to increase, with a peak at 16 h, reaching 16.4% (d.w.), equivalent to 94.7% of the maximum theoretical yield (Table 2).

**TABLE 2:** Content of sugars and dextran during the fermentation of BSG in conditions without (EPS‐BSG) and with (EPS + BSG) sucrose addition and dextran synthesis (values are expressed as average % d.w.).

Time points	Galactose	Glucose	Sucrose	Fructose	Maltose	Dextran
**EPS‐BSG**						
T0	nd	3.3 ± 0.8^a^	nd	0.1 ± 0.01^a^	1.2 ± 0.2^a^	nd
T8		2.4 ± 0.2^a^		0.08 ± 0.01^b^	1.4 ± 0.1^a^	
T10		0.7 ± 0.1^b^		0.09 ± 0.01^b^	1.2 ± 0.1^a^	
T16		nd		nd	0.2 ± 0.02^b^	
T24					nd	
**EPS + BSG**						
T0	nd	3.3 ± 0.8^a^	34.6^a^ *	0.1 ± 0.01^a^	1.2 ± 0.2^a^	nd
T8	0.1 ± 0.02^a^	0.5 ± 0.01^b^	14.2 ± 1.8^b^	11.5 ± 0.3^b^	0.7 ± 0.03^b^	8.5 ± 0.6^a^
T10	0.2 ± 0.03^b^	0.6 ± 0.02^b^	6.5 ± 0.4^c^	12.3 ± 1.1^b^	0.9 ± 0.03^c^	11.9 ± 0.4^b^
T16	nd	0.9 ± 0.05^c^	nd	13.2 ± 0.4^c^	0.7 ± 0.02^b^	16.3 ± 0.4^c^
T24		1.6 ± 0.03^d^		10.9 ± 0.4^b^	0.4 ± 0.01^d^	16.4 ± 0.2^c^

*Note:* Values in the same column of each fermenting conditions (EPS‐BSG and EPS + BSG) with different letters are significantly different (*p* < 0.05).

Abbreviation: nd, not detected.

* Sucrose was added for EPS + BSG fermentation.

At T0, BSG samples contained 3.3% glucose, 0.1% fructose, and 1.2% maltose, while xylose, galactose, and sucrose (in EPS‐BSG) were not detected (Table 2). During fermentation of EPS‐BSG, the glucose level decreased from the initial value to 0.7% at T10, and it was not detectable anymore at the later time points (Table 2). Fructose was detectable within the first 10 h with levels below 0.1% and was not detected at later time points. Galactose and sucrose were not detected during fermentation, while maltose remained unchanged until 10 h of fermentation, after which it declined to 0.2% at 16 h and was not detectable anymore at 24 h.

Notable differences were observed in the EPS + BSG fermentation process with added sucrose (Table 2). Glucose rapidly declined within the first 8 h, from 3.3% to 0.5%, after which there was an increase towards the end of the fermentation, reaching 1.6% at 24 h. Low levels of galactose were detected at 8 and 10 h, while maltose decreased from 1.2% at T0 to 0.4% at 24 h. The added sucrose (initially 34.6%) was rapidly converted to dextran and fructose, with approximately 57% utilised within the first 8 h, 81% after 10 h (14.2% and 6.5% at T8 and T10, respectively). No sucrose was detected from 16 h onwards. The fructose levels increased from 0.1% at T0 to 11.5% after 8 h and reached a maximum level at 16 h (13.2%) after which it decreased to 10.9% at 24 h (Table 2). Notably, a substantial amount of mannitol (15.5% d.w.) was formed in the EPS + BSG fermentation, which was detected only in the 24 h sample.

**FIGURE 1:**
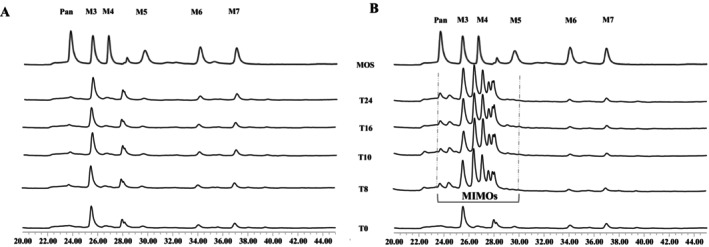
Oligosaccharide profile of fermented BSG in (A) EPS‐BSG (without sucrose addition) and (B) EPS + BSG (with sucrose addition). The horizontal axis of the chromatograms represents the retention time (min). The maltooligosaccharide reference standards are indicated as follows: Pan (panose) at 24 min, M3 (maltotriose) at 25.5 min, M4 (maltotetraose) at 27 min, M5 (maltopentaose) at 30 min, M6 (maltohexaose) at 34 min, and M7 (maltoheptaose) at 37 min.

There was a clear difference in the oligosaccharide profiles when BSG was fermented with or without the added sucrose (Figure 1). The oligosaccharide profile remained consistent throughout the fermentation of EPS‐BSG and mainly consisted of maltooligosaccharides. However, during the fermentation of EPS + BSG, a significant increase of MIMO eluting between panose and maltopentaose (Figure 1) was observed (T8‐T24h).

### 3.3 Key Outcomes of the Transcriptome Analysis

**FIGURE 2:**
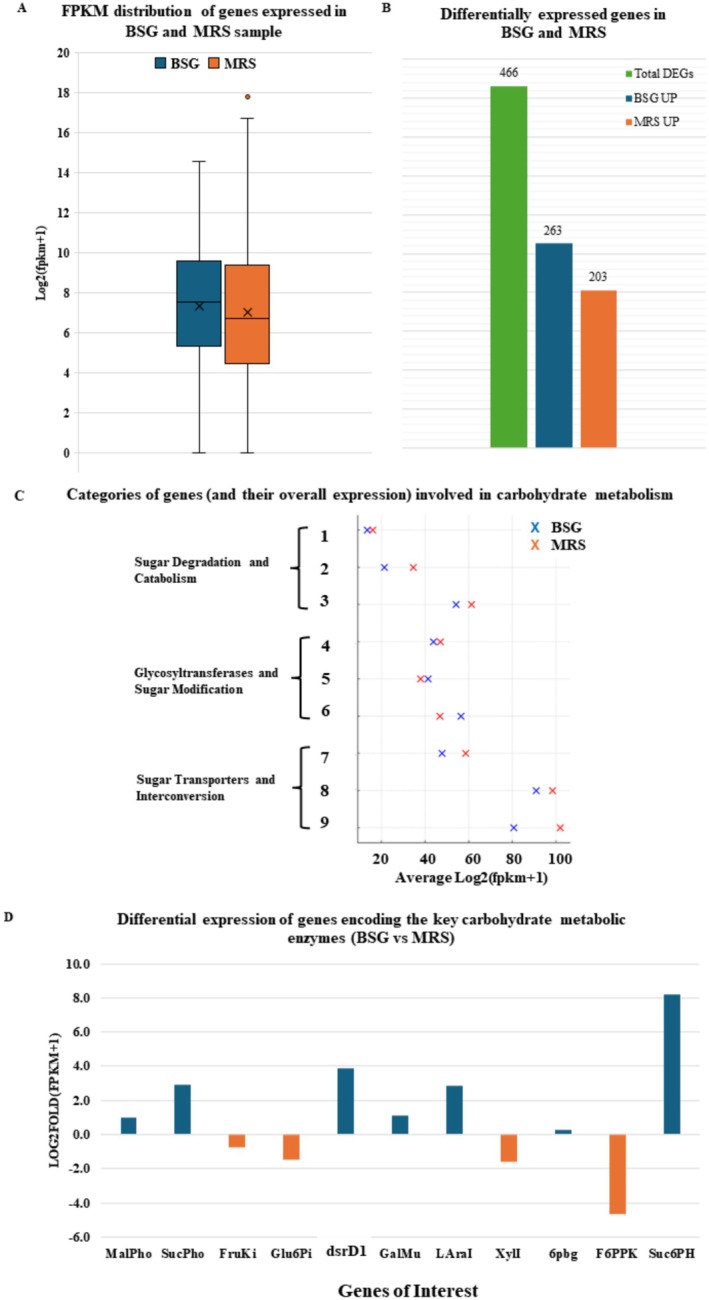
Overview of the transcriptome analysis outcomes highlighting the carbohydrate metabolism relevant for the BSG fermentation. Analysis of differential gene expression between 24‐h MRS cultivation (equivalent to T0 of the BSG fermentation) and 16‐h ESP + BSG fermentation. (A) The box plot shows the distribution of gene expression levels between BSG and MRS samples (the boxes represent the interquartile range (IQR), the line inside each box shows the median, whiskers extend to 1.5 times the IQR, dots represent outliers, and ‘x’ marks indicate the mean values). (B) The bar chart showing the number of differentially expressed genes between BSG and MRS samples. (C) The dot plot illustrating the overall level of expression of different functional groups of genes involved in carbohydrate metabolism under BSG and MRS conditions (1: maltose phosphorylases, 2: sucrose hydrolases and phosphorylases and fructose bisphosphatases, 3: dTDP‐glucose, phosphoketolase, galactokinase, and G6PDH, 4: sucrose, maltose hydrolases and UTP/UDP‐glucose enzymes, 5: lipopolysacchardie and peptidoglycan biosynthesis, 6: glycosyltransferase family proteins, 7: xylose isomerases and arabinose transporters, 8: isomerases and mutarotase enzymes: glucose, galactose, and phosphate transfer, and 9: PTS system components). (D) The bar chart depicts the fold changes in expression (ESP + BSG T16 vs. MRS 24 h ≈ BSG T0) of selected individual genes encoding the enzymes relevant to the metabolism of carbohydrates as monitored in the BSG fermentations.

To better understand the carbohydrate metabolism during BSG fermentation and dextran synthesis, the whole genome transcriptome was analysed and compared between the two main stages of this process: 24 h cultivation in MRS—representing T0 of the BSG fermentation, i.e., the cells before the inoculum in BSG—and 16 h of BSG fermentation—representing the advanced adaptation stage of *L. pseudomesenteroides* to this environment. On a general level, the FPKM (fragments per kilobase of transcript per million mapped reads) distribution of genes (Figure 2A) expressed in both BSG and MRS samples showed similar adjusted mean expression values. However, the median gene expression level was higher in MRS than in BSG, which indicates decreased variability in gene expression in MRS, as shown by the narrower interquartile range indicating a more consistent gene expression profile. A total of 466 genes showed significant differences in expression between BSG and MRS samples (Figure 2B). Of these, 263 genes were expressed higher in BSG, while the expression of 203 genes was higher in MRS. The expression level of genes (Figure 2C) involved in carbohydrate metabolism varied significantly between the two types of samples. The phosphotransferase system (PTS) components had higher average expression in MRS, indicating a robust activity of the carbohydrate uptake system. Although there was slightly higher expression in MRS, isomerases, and mutarotase enzymes, crucial for carbohydrate metabolism, were highly expressed in both conditions.

Genes specifically involved in sucrose metabolism, such as SucPho (sucrose phosphorylase), dsrD1 (dextransucrase), and Suc6PH (sucrose‐6‐phosphate hydrolase), had higher expression levels during fermentation of BSG when compared to MRS. This is consistent with the enhanced utilisation of sucrose observed during the fermentation of EPS + BSG. Conversely, genes involved in fructose and glucose metabolism, such as FruKi, Glu6Pi, XylI and F6PPK (fructokinase, glucose‐6‐phosphate isomerase, xylose isomerase and fructose‐6‐phosphate phosphoketolase), were expressed at higher levels in MRS, which highlights intricate and complex metabolic regulation in these conditions.

Very significant gene expression differences between the two tested conditions were associated with carbohydrate sensing, signalling and transport. Several PTS and other PTS‐like transporters involved in sugar uptake showed differential expression levels. In the BSG condition, genes like alpha‐amylase, beta‐glucoside transporter subunit IIABC, and sugar transporter subunit IIA had reduced expression levels. Conversely, other PTS and PTS‐like components, such as several membrane proteins and the beta‐glucoside transporter subunit EIIBCA, showed higher expression, indicating that PTS‐mediated sugar transport systems, including the phosphorylation of sugars during transport across the cell membrane, were more active during the BSG fermentation. Numerous transcriptional regulators, including the Crp/Fnr and GntR families, exhibited variable expression levels, suggesting dynamic regulatory events occurring in the BSG fermentation.

In the T16 EPS + BSG sample, ten genes related to carbohydrate metabolism and membrane transport were upregulated, indicating their roles in carbohydrate catabolism. These genes include MFS transporter proteins, which are crucial for sugar transport across cell membranes. Additionally, the alpha‐ketoglutarate transporter, essential for translocating various sugars, including glucose, fructose, and mannose, across the membrane, was upregulated. The membrane protein‐PTS, responsible for both the transport and phosphorylation of sugars for further metabolism, and sucrose‐6‐phosphate hydrolase, which hydrolyses sucrose‐6‐phosphate into fructose‐6‐phosphate and inorganic phosphate, were also notably expressed in the T16 EPS + BSG sample. Conversely, significantly lower expression of 21 genes related to carbohydrate metabolism and transport was observed in the T16 EPS + BSG sample. These include genes encoding essential enzymes and transporters such asbeta‐D‐galactosidase, which hydrolyzes beta‐glycosidic bonds to release glucose and galactose during cellulose and hemicellulose metabolism; fructokinase, which catalyses the phosphorylation of fructose to fructose‐1‐phosphate; glucose‐6‐phosphate isomerase, which facilitates the isomerization of glucose‐6‐phosphate to fructose‐6‐phosphate—an essential reaction in both glycolysis and gluconeogenesis; mannose‐6‐phosphate isomerase, part of the PTS system specific for the transport of mannose, fructose, and sorbose; phosphoketolase, involved in breaking down pentoses and hexoses into pyruvate and acetyl phosphate; and the PTS beta‐glucoside transporter subunit IIABC, responsible for the uptake and phosphorylation of beta‐glucosides critical for cellulosic and hemicellulosic carbohydrate metabolism, which were downregulated in the T16 EPS + BSG.

**FIGURE 3:**
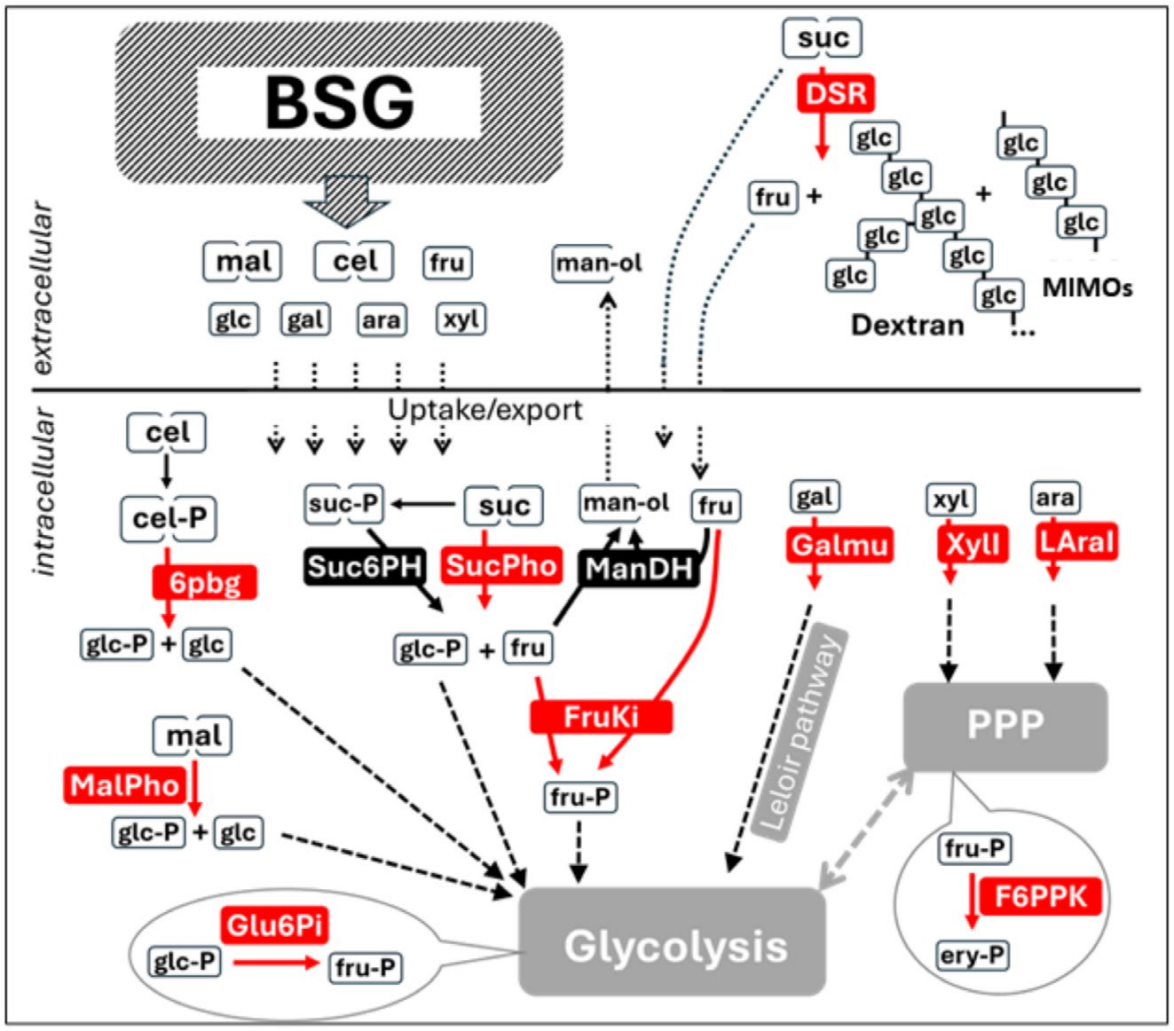
Simplified overview of the key carbohydrate conversions during the *L. pseudomesenteroides* fermentation of BSG with added sucrose. The BSG is a source of diverse carbohydrates released from starch and lignocellulose via enzymatic activities during the brewing process and during the fermentation. The sugars released from BSG include disaccharides—such as maltose (mal) or cellobiose (cel), and monosaccharides—such as glucose (glu), fructose (fru), arabinose (ara), xylose (xyl), or galactose (gal). The extra sucrose (suc) added to the fermentation was the main substrate for production of dextran, fructose, and MIMOs (maltosylisomaltooligosaccharides), which were synthetized in the extracellular space by the action of dextransucrase(s) (DSR) secreted by *L. pseudomesenteroides* during fermentation. The enzymes shown in the figure (in red) have been analysed in this study by monitoring their gene expression during a time‐course of ESP + BSG and ESP‐BSG fermentations, and their selection was motivated by their significance to the BSG‐related carbohydrate metabolism. The enzymes that are involved in the metabolism of sugars derived from BSG include: 6‐phospho‐beta‐glucosidase (6pbg), maltose phosphorylase (MalPho), galactose mutatorase (GalMu), xylose isomerase (XylI), and L‐arabinose isomerase (LAraI). The enzymes involved in metabolism related to sucrose and fructose include sucrose phosphorylase (SucPho), sucrose‐6‐phosphate hydrolase (Suc6PH), fructokinase (FruKi), and mannitol dehydrogenase (ManDH) responsible for formation of mannitol (man‐ol) from fructose. Other enzymes involved in essential metabolic reactions include glucose‐6‐phosphate isomerase (Glu6Pi), and fructose‐6‐phosphate phosphoketolase (F6PPK) associated with pentose phosphate pathway (PPP) and responsible for sugars interconversions, such as fructose‐6‐phosphate (fru‐P) to erythrose‐4‐phosphate (ery‐P) required for balancing intracellular carbohydrate pools.

Specific attention was given to the genes encoding enzymes directly involved in the sugar metabolism relevant to the EPS + BSG fermentation (Figures 2D and 3). The analysis revealed that at 16 h of the EPS + BSG fermentation, there was higher relative expression of genes encoding maltose phosphorylase (Malpho), sucrose phosphorylase (Sucpho), sucrose‐6‐phosphate hydrolase (Suc6PH), galactose mutarotase (Galmu), L‐arabinose isomerase (LaraI), 6‐phospho‐beta‐glucosidase (6pbg) and dextransucrase previously identified as dsrD1 (Koirala et al. 2021). On the contrary, other genes also associated with the BSG fermentation showed lower relative expression in EPS + BSG. These were fructokinase (FruKi), glucose‐6‐phosphate isomerase (Glu6Pi), xylose isomerase (Xyli) and fructose‐6‐phosphate phosphoketolase (F6PPK).

Two mannitol dehydrogenase genes were identified as significantly more expressed in EPS + BSG than in MRS, with log2 FoldChange values of 3.8 and 4.5 and corresponding p‐values indicating high statistical significance. Additionally, two novel genes were identified, containing strong homology to alcohol dehydrogenases. These findings suggest high activity of mannitol or alcohol dehydrogenases during BSG fermentation, and in the case of the mannitol dehydrogenases, they correlate with the substantial amounts of mannitol detected at 24 h of the EPS + BSG fermentation. Mannitol dehydrogenase is responsible for the conversion of fructose to mannitol in the heterofermentative LAB (Martínez‐Miranda et al. 2022).

During the analysis of the transcriptome sequences, six glycosyltransferase‐encoding genes with a significant sequence homology to the known dextransucrases were identified in the genome of *L. pseudomesenteroides* DSM20193, but only one showed significantly higher expression at 16 h of EPS + BSG fermentation as compared to MRS. The dsrD1 gene showed the induction with a 3.9 Log2Fold(fpkm+1) change in BSG vs. MRS. Amongst the putative dsrD genes identified, the two previously studied, dsrD2 and dsrD3 (Koirala et al. 2021), were also identified. Apart from dsrD1, one other dsrD‐related gene showed different expression levels between the BSG and MRS conditions, and their possible contribution to dextran production remains unclear.

### 3.4 Targeted Transcription Analysis of Dextran‐ and Sugar Metabolism‐Related Genes

Based on the above analyses, the sugar content determination (Table 2) and especially the transcriptome results, ten genes were selected (Figure 3) for targeted comparative transcription analysis during EPS + BSG and EPS‐BSG fermentations in a time‐course manner. These genes were maltose phosphorylase (Malpho), sucrose phosphorylase (Sucpho), sucrose‐6‐phosphate hydrolase (Suc6PH), galactose mutarotase (Galmu), L‐arabinose isomerase (LaraI), 6‐phospho‐beta‐glucosidase (6pbg), dextransucrase (dsrD1), fructokinase (FruKi), glucose‐6‐phosphate isomerase (Glu6Pi), xylose isomerase (Xyli) and fructose‐6‐phosphate phosphoketolase (F6PPK). The main aims were to (1) confirm the findings of the transcriptome analysis, (2) investigate the expression in different phases of the fermentations, and (3) elucidate differences in the carbohydrate metabolism of *L. pseudomesenteroides* in BSG with and without sucrose addition. The results are summarised in Figure 3.

**FIGURE 4:**
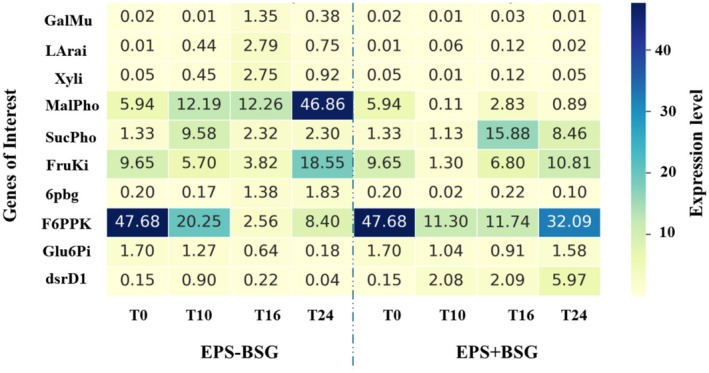
Relative expression levels of the *L. pseudomesenteroides* DSM20193 genes related to carbohydrate metabolism during different stages (T0, T10, T16, T24) of the BSG fermentation with (EPS + BSG) or without (EPS–BSG) without added sucrose. The values represent expression levels normalised (relative) to the expression of the recA gene.

In the 24‐h fermentation of EPS‐BSG, the dextransucrase gene, dsrD1, showed negligible expression. However, when BSG was fermented with sucrose (EPS + BSG), the dsrD1 expression significantly increased at 10 h (14‐fold from T0) and peaked at 24 h (40‐fold from T0), indicating that dsrD1 remained highly active during all 24 h of the EPS + BSG fermentation, which correlates with the viscosity and dextran accumulation during EPS + BSG fermentation (Tables 1 and 2). Different expression patterns were observed for some sugar metabolism‐related genes between EPS− and EPS + BSG fermentation conditions, indicating a significant impact of the sucrose addition in BSG. For instance, MalPho expression increased at 10 h in both conditions; however, it continued increasing progressively during the EPS‐BSG fermentation, showing the highest expression at 24 h, whereas its expression declined substantially in the later stages of EPS + BSG fermentation. The expression of Galmu, LaraI, XylI and 6pbg—representing genes for alternative sugar metabolic enzymes—was negligible throughout the EPS + BSG fermentation, while their expression increased or remained stable during the EPS–BSG. This may be explained by a need for the alternative carbon sources available in BSG in the condition where glucose or other readily metabolised sugar (such as sucrose) is limiting. SucPho expression transiently increased in T10 of the EPS‐BSG cultivation, while in EPS + BSG, its expression significantly increased at T16 and remained relatively high until 24 h. FruKi expression mildly decreased at T10 in both conditions and increased again towards the end of the fermentations, with more significant upregulation at T24 in EPS–BSG. F6PPK2 showed the highest expression in T0 (MRS), followed by a decreased expression in both conditions, which was however regained at the last timepoint of the EPS + BSG fermentation. The expression of Glu6Pi remained relatively stable throughout the EPS + BSG fermentation, but it showed a progressive decrease in EPS‐BSG. In summary, the overall expression pattern observed in the EPS‐BSG samples indicated numerous metabolic adaptations to the condition with less available alternative carbon courses released from BSG, whereas the expression pattern in EPS + BSG was strongly influenced by the abundant amounts of sucrose, fructose, and, to a lesser extent, glucose (Table 2 and Figure 4).

## 4 Discussion

In this study, we investigated how different sugars present in BSG are metabolised during fermentation by *L. pseudomesenteroides* DSM20193 in conditions with and without dextran synthesis. LAB fermentation has proven effective in valorising this by‐product (Bianco et al. 2020; Koirala et al. 2021; Neylon et al. 2021b). However, understanding how fermentation progresses in the presence of multiple and competitive carbon sources would be beneficial in maximising the potential of LAB in transforming complex substrates like BSG into a product of value for the food chain. In controlled fermentations, LAB reached ca. 9.2 Log CFU/g after 24 h in both EPS‐BSG and EPS + BSG samples, showing an increase of ca. 3 log cycles, while after 24 h of spontaneous fermentation, the presumptive LAB cell density remained the same as in the native BSG, i.e., < 3 Log CFU/g. This indicated that very low competition occurred due to indigenous LAB microbiota. The pH drop in EPS‐BSG was slower than in EPS + BSG, showing a variation of 1.7 and 2.1 units after 24 h, respectively. This result aligns with the rest of the acidification parameters and with previous studies, showing higher TTA and lactic and acetic acid levels in the condition of dextran synthesis (Koirala et al. 2021, 2022; Xu et al. 2018, 2019).

Dextran yield is quantitatively defined as the proportion of dextran produced during fermentation relative to the sucrose substrate used, with the theoretical maximum yield of dextran being 50% of the added sucrose (Katina et al. 2009; Kothari et al. 2015). In our previous study, we observed a theoretical dextran yield of 60% during fermentation of BSG with the same starter (Koirala et al. 2021), which was significantly lower than in this study, i.e., 94%. The dextran content is important as it influences the viscosity of EPS + BSG and enhances its performance as a food ingredient. Typically, a dextran yield of 40%–60% has been obtained during LAB fermentation of different plant‐based materials such as BSG, chickpea, sorghum, faba bean, and pea flour (Galli et al. 2020; Koirala et al. 2021, 2022; Perri et al. 2021; Shuai et al. 2023; Wang et al. 2018, 2020; Wang, Maina, et al. 2021; Wang, Wu, et al. 2021). In all these cases, the amount of dextran produced effectively acted as a hydrocolloid with a positive technological impact. The higher dextran yield in this study compared to previous studies can be attributed to several factors, such as primarily the composition of BSG, which is affected by different brewing practices (Naibaho and Korzeniowska 2021; Santos et al. 2003). Notably, the BSG used in our prior study (Koirala et al. 2021) contained 4.4% more dry matter and 7.4% more dietary fibre but had a 3.6% lower protein content compared to the BSG in the current study. While the sucrose to BSG ratio remained constant, the water to BSG ratio was slightly higher in this study (61.5% vs. 60%). However, this minimal difference in water is unlikely to have a significant impact on the fermentation dynamics and dextran production.

Our data shows that viscosity increased steadily over the 24‐h fermentation period. Meanwhile, the amount of synthesised dextran increased up to 16 h and then remained stable for the rest of the fermentation period. This suggests that factors other than just the concentration of dextran could affect the change in viscosity. Viscosity is influenced by multiple components in the fermentation medium, and the contribution of dextran to viscosity is not linear. The actual viscosity value is determined by the molecular weight distribution, solubility, and the interaction of polymers with other components present in the fermentation medium, BSG in this case (Nácher‐Vázquez et al. 2017). The stability or constant quantity of dextran synthesis after 16 h indicates that the synthesis capacity may have reached the maximum, possibly due to nutrient depletion (Federici et al. 2014). It is possible that structural rearrangement and further polymerisation of existing dextran molecules lead to an increase in molar mass, which can enhance the degree of intermolecular entanglement and, consequently, an increase in viscosity (Tohno et al. 2012). Furthermore, the release of macromolecules such as polysaccharides and proteins during the later stage of fermentation may also contribute to the increase in viscosity (Koirala et al. 2021; Harris and Smith 2006; Qi et al. 2023).

The oligosaccharides profile remained similar throughout the fermentation of EPS‐BSG. Most of the peaks belonged to DP3, DP4 and DP5 (compared to reference standards: maltotriose, maltotetraose and maltopentaose); a similar trend was also observed earlier (Koirala et al. 2021). In the current study, however, oligosaccharides with a DP7 were detected at T0 and persisted throughout the fermentation, indicating they were native to the used BSG. Meanwhile, during the fermentation of EPS + BSG, MIMO were synthesised concomitantly with dextran due to the action of the acceptor reactions by dextransucrase (Jung and Mayer 1981; Robyt et al. 2008), mainly using maltose as an acceptor (Galle and Arendt 2014; Grewal and Khare 2018; Leemhuis et al. 2013).

The comparative analysis of sugar metabolism during EPS + BSG fermentation by *L. pseudomesenteroides* DSM20193 across this and previous studies (Koirala et al. 2021) explained the dynamics of sugar consumption and its adaptive nature in different environmental conditions. Glucose was rapidly consumed in both studies, with the current study showing even faster consumption, indicating an enhanced metabolic response by the starter. This could be due to the release of glucose from complex carbohydrates during fermentation by the action of endogenous carbohydrases (De Vos 1996; Koirala et al. 2021; Tarraran and Mazzoli 2018), which occurred more rapidly in the current study than observed previously. Endogenous enzymes in BSG include carbohydrate‐degrading enzymes such as xylanases, cellulases, feruloyl esterases, and proteases (Lynch et al. 2021). The activities of these endogenous enzymes are significantly affected by the pH value, with optimal activities occurring around pH 5.0–5.5 for carbohydrate‐degrading enzymes and around pH 6.0 for proteases. The pH levels from T0 to T10 remained between 5.0 and 6.0 when BSG was fermented with and without added sucrose, suggesting that these endogenous enzymes may have been active for a significant time during the total 24‐h fermentation period. This stable pH was most likely contributing to the continuous release of glucose and maltose during fermentation, which were also steadily utilised as fermentation progressed.

Sucrose levels decreased significantly by T8 in the current study, yet remained detectable until T10, unlike previously observed (Koirala et al. 2021). The rapid decline of sucrose in both studies underscores its importance as a preferred energy source and a substrate for dextran synthesis. Fructose exhibited relatively stable, high concentration across time points T8, T10, T16, and T24 during EPS + BSG, likely due to its simultaneous release by dextransucrase and use as a carbon source for metabolism (Robyt et al. 2008). In EPS‐BSG, the amount of fructose was gradually depleted after 16 h.

Maltose concentration increased throughout the fermentation process, peaking at T16 before experiencing a slight decrease by T24, suggesting the breakdown of complex sugars into maltose via residual endogenous enzymes of BSG and its consumption. In the absence of sucrose, glucose, fructose, and maltose were rapidly metabolised, leading to their complete depletion at 24 h. The fermentation outcome in both conditions suggests an efficient utilisation of the available substrates, showing an adaptable response to the fermentation environment.

Carbohydrate metabolism was investigated using a transcriptomic approach (RNA sequencing) as well as gene‐specific transcription analysis. Initially, whole genome expression was studied through RNA sequencing in two different growth conditions: (1) at the beginning of the fermentation process and (2) after 16 h of BSG fermentation in the presence of sucrose (EPS + BSG). The first condition represented the status of cells after cultivation in MRS broth for 24 h at 30°C, i.e. the cells at the inoculum point. This condition was expected to exhibit increased expression of genes associated with sustained metabolic activity, nutrient utilisation, and prolonged survival, highlighting the starter response to an enriched and controlled environment (Liu et al. 2020; Papadimitriou et al. 2016). The second condition represented the cells adapted to BSG and showing high dextransucrase activity, as observed in our previous study (Koirala et al. 2021). Transcriptome analysis revealed the metabolic pathways regulated in MRS broth and BSG fermentation environments. Cells grown in MRS exhibited elevated levels of expression of genes related to pathways linked to ABC sensing and teichoic acid production, indicating a greater activity of cellular transport and signalling processes (Papadimitriou et al. 2016). Compared to growth in MRS, in EPS + BSG fermentation, genes associated with maltose, sucrose, dextransucrase, galactose, L‐arabinose, and galactose metabolism showed higher expression (with relative differences ranging from 1.2 to 14.8‐fold). In contrast, genes related to fructose, glucose, and xylose metabolism were less expressed (relative differences of 0.33 to 0.59‐fold). The expression of genes involved in the PTS and pyruvate metabolism was different in the two conditions, indicating potential differences in sugar transport and energy metabolism. Cells grown in MRS had higher expression levels associated with key biological activities such as bacterial cell wall synthesis, sugar metabolism, mannose, fructose, and sorbose metabolism. Finally, higher expression of genes related to processes involving the interconversion of UDP‐galactose and UDP‐glucose was observed in the MRS samples, indicating functional specialisation in fermenting MRS in the context of sugar metabolism and transport (Reizer et al. 1988; Thompson 1987). This information would suggest a specific gene response in BSG, which may reflect adaptations to its unique nutritional environment. Specifically, genes related to the catabolism of carbohydrates native to BSG (Ikram et al. 2017; Jin et al. 2022; Mussatto et al. 2006), such as Malpho, FruKi, Glu6Pi, Galmu, Larai and Xyli for maltose, fructose, glucose, galactose, arabinose and xylose, respectively, were selected to monitor their regulation at different time points during BSG fermentation. Additionally, Sucpho and DSR genes encoding sucrose phosphorylase and dextransucrase, along with 6pbg and F6PPK genes to target 6‐phospho‐beta‐glucosidase and fructose‐6‐phosphate phosphoketolase specifically for complex carbohydrate metabolism (Acin‐Albiac et al. 2021; Zhang, Wang, et al. 2023), were selected to observe their expression patterns at different time points during BSG fermentation via qPCR.

The sugar metabolism dynamics during EPS + BSG and EPS‐BSG fermentation revealed preferential substrate utilisation and dextransucrase‐mediated pathways, as demonstrated by a decrease in glucose amount in EPS‐BSG and a significant decrease in sucrose with an increasing amount of dextran produced in EPS + BSG. During the fermentation process, as expected, there was a noticeable increase in the expression of the DSR gene in the EPS + BSG condition compared to EPS‐BSG at T10 and T16. By T24, the highest expression of the dextransucrase gene was observed in the EPS + BSG condition; however, the amount of dextran had already reached its highest level at T16. The dsrD1 gene remained active throughout the fermentation period, even after all the sucrose had been metabolised and utilised to produce the maximum amount of dextran possible. The sustained activity of the dextransucrase gene beyond the complete utilisation of sucrose suggests that this gene might remain active despite sucrose availability due to environmental factors and the tendency to utilise alternative carbon sources (Vallejo‐García et al. 2023). In this study, it was found that although multiple DSR genes are present in *L. pseudomesenteroides* DSM20193, only one was active and responsible for dextran synthesis. However, in our first study, this gene had its highest expression at T10 and decreased to a lower level after 24 h, unlike in the present conditions. Differences in the expression of DSR genes could be due to differences in the environment, especially related to the varying compositions of BSG (Koirala et al. 2021).

A significant increase in expression was observed, especially for the Sucpho gene at T16 during EPS + BSG fermentation, correlating with active sucrose metabolism when compared to EPS –BSG fermentation. During the fermentation of EPS + BSG, dextransucrase was induced, leading to fructose release and dextran synthesis (Robyt et al. 2008).

During the fermentation of EPS‐ and EPS+ BSG, the expression of F6PPK appeared to be influenced by the presence of fructose. F6PPK expression initially decreased during EPS + BSG fermentation despite the increase in fructose levels but significantly increased at 24 h, indicating the involvement of additional regulatory factors, whereas its expression steadily declined in EPS‐BSG due to the absence of fructose (Zotta et al. 2018). With no fructose to directly control the F6PPK expression, the high expression of F6PPK during the growth in MRS and the early stage of EPS‐BSG fermentation could be due to the need to utilise alternative carbon sources in these environments. LAB adjust their metabolic pathways to utilise complex carbohydrates in the presence of plant biomass, and this shift is critical for their growth, survival, and the progression of fermentation (Gaspar et al. 2013; Septembre‐Malaterre et al. 2018). CCR is essential in determining the preferential utilisation of carbon sources in LAB during fermentation, considerably altering metabolic pathways and enzyme expression to adapt to changing environments (Görke and Stülke 2008). In the presence of favoured carbon sources such as glucose, CCR can trigger decreased expression of genes encoding enzymes such as F6PPK, a critical compound in the pentose phosphoketolase pathway (Görke and Stülke 2008; Stülke and Hillen 1999). When easily metabolised carbon sources are present, the expression of 6pbg and genes involved in alternate sugar metabolism are inhibited (Brückner and Titgemeyer 2002; Chen et al. 2018; Mahr et al. 2000).

The observed gene expression patterns underscore the distinct functional roles of the enzymes in sugar metabolism under varying fermentation conditions. In the context of sugar metabolism during dextran formation, the activity of the dextransucrase enzyme, particularly the dsrD1 gene, plays a critical role. This process is crucial in EPS + BSG conditions where sucrose is abundantly available, as evidenced by the significant upregulation of dsrD1. The complementary activities of sucrose‐6‐phosphate hydrolase and sucrose phosphorylase ensure that the products of sucrose hydrolysis are efficiently channelled into metabolic pathways that support both energy production and biosynthesis, enhancing the efficiency of dextran synthesis by dextransucrase. Similarly, the expression of FruKi (fructokinase) in EPS + BSG suggests that the released fructose is efficiently phosphorylated to fructose‐6‐phosphate, integrating into the glycolytic pathway and facilitating continuous energy production and cellular functions. In addition, the upregulation of genes encoding mannitol dehydrogenase (ManDH) correlates with the metabolic route of fructose conversion to mannitol, which accumulated at the end of EPS + BSG fermentation. These observations highlight a well‐coordinated metabolic shift towards utilising sucrose for dextran production and maintaining metabolic balance through fructose metabolism. In contrast, the fermentation of BSG without added sucrose (EPS‐BSG) showed higher expression of genes involved in the metabolism of alternative sugars such as galactose, arabinose, and xylose. This indicates that in the absence of excess sucrose, *L. pseudomesenteroides* DSM20193 adapted by utilising available sugars through pathways involving GalMu (galactose mutatorases), Larai (L‐arabinose isomerase), and Xyli (xylose isomerase) enzymes. The substantial expression of MalPho (maltose phosphorylase) at later stages in EPS‐BSG suggests delayed but significant maltose metabolism, while 6pbg (6‐phospho‐beta‐glucosidase) facilitated the breakdown of beta‐glucosides, showing varied activity depending on the fermentation conditions. The correlation between sugar consumption and gene expression during EPS + BSG and EPS‐BSG fermentation processes further confirmed the adaptation mechanisms of *L. pseudomesenteroides* DSM20193. During the course of the EPS‐BSG fermentation, gradual expression changes of Malpho, Sucpho, FruKi, F6PPK2, Xyli, Larai, and GalMu indicate dynamic metabolic shifts in the utilisation of diverse available sugars. On the other hand, in EPS + BSG, a very limited tendency of shifting to alternative sugars metabolism was observed in the cultivation with a mild increase of Malpho expression, corresponding to slows maltose utilisation observed during the fermentation. Fructose, released during dextran synthesis, and its varying consumption patterns align with the differential expression of FruKi, ManDH and F6PPK, highlighting the adaptation to available fructose levels.

This study is the first to investigate the differences in the utilisation of sugars and gene expression in conditions of dextran synthesis during fermentation of a complex medium consisting of a mixture of BSG, water and sucrose. This study showed a greater metabolic flexibility and an adaptive response to the BSG substrate of *L. pseudomesenteroides* DSM20193 compared to growth in MRS.

## 5 Conclusions

Our study demonstrated the metabolic differences in the preferred utilisation of simple and complex sugars by *L. pseudomesenteroides* DSM20193 during adaptation to a complex food environment like BSG. The differential preference of sugars during the fermentation of BSG in dextran and oligosaccharides synthesis conditions was evidenced by the absence of expression of genes involved in alternative sugar metabolism and the induction of genes relevant for sucrose and fructose utilisation. The differential gene responses demonstrated a significant upregulation of sucrose and maltose metabolism genes and a downregulation of genes related to alternative carbon source metabolism, reflecting the strain's efficient adaptation to optimise dextran production in complex food environments.

Understanding metabolic pathways and genetic adaptations during BSG fermentation with LAB can help tailor the process to develop fermented raw materials with specific properties. This approach enables the effective use of BSG and other side streams as functional ingredients, expanding the use of diverse sugars beyond glucose. LAB fermentation presents a viable method for valorising agro‐industrial side streams like BSG, with significant implications for sustainable food production. Further optimisation of fermentation conditions could enhance dextran yield and explore the functional properties of fermented BSG for various food applications.

## Author Contributions

**Koirala Prabin:** investigation, methodology, writing – review and editing, writing – original draft, conceptualization, visualization, validation, formal analysis, data curation. **Maina Ndegwa:** methodology, validation, writing – review and editing. **Mojzita Dominik:** writing – original draft, visualization, methodology, formal analysis, validation, conceptualization. **Coda Rossana:** conceptualization, writing – original draft, funding acquisition, methodology, project administration, supervision, resources, validation, formal analysis.

## Conflicts of Interest

The authors declare no conflicts of interest.

## Supporting information


**Table S1.** RNASeq library preparation, sequencing, data analysis methods and data (raw and analysed).
**Table S2.** Target genes and primers used in RT‐qPCR.
**Table S3.** qPCR run protocol.

## Data Availability

The data that support the findings of this study are openly available in OneDrive – University of Helsinki at https://helsinkifi‐my.sharepoint.com/my?id=%2Fpersonal%2Fkoirala%5Fad%5Fhelsinki%5Ffi%2FDocuments%2FUni%20desktop%2FRNA%20seq%20data%5F21032023;ga=1.
