# Supplementary material for: Regulation of Sugar Metabolism During Fermentation of Brewers' Spent Grain by Leuconostoc pseudomesenteroides DSM20193
Source: Microb Biotechnol. 2025 Apr 21;18(4):e70116. doi: 10.1111/1751-7915.70116 (PMC12010140; doi:10.1111/1751-7915.70116)
Supplement: Supplementary file 1 — Table S1. RNASeq library preparation, sequencing, data analysis methods and data (raw and analysed). Table S2. Target genes and primers used in RT‐qPCR. Table S3. qPCR run protocol. [file MBT2-18-e70116-s001.docx]

**Supplementary information**

**Regulation of sugar metabolism during fermentation of brewers’ spent grain by *Leuconostoc pseudomesenteroides* DSM20193**

**Authors:** Koirala Prabin^1,*^, Maina Ndegwa^1^, Mojzita Dominik^2^, Coda Rossana^1,3^

^1^ Department of Food and Nutrition, University of Helsinki, P.O. Box 66 (Agnes Sjöbergin Katu 2), 00014 Helsinki, Finland

^2^ VTT Technical Research Centre of Finland Ltd, P.O. Box 1000, FI-02044 VTT, Finland

^3^ Faculty of Agriculture and Forestry, Helsinki Institute of Sustainability Science (HELSUS), University of Helsinki, 00014 Helsinki, Finland

**^*^Corresponding author**: Koirala Prabin ([prabin.koirala@helsinki.fi](mailto:prabin.koirala@helsinki.fi))

**Table S1: RNASeq library preparation, sequencing, data analysis methods and data (raw and analyzed)**

| **Heading** | **Links to get access** |
| --- | --- |
| Library preparation, Sequencing, and data (incl. statistics) method | [Sequencing_method_Novogene.pdf](https://helsinkifi-my.sharepoint.com/:b:/g/personal/koirala_ad_helsinki_fi/ER_r_1nWlotMt_up_hjcV5gBu7d4ZlpDkCYNAo_BGzXJ9g?e=V4iPjr)  [Methods of Data Analyses_Novogene_RNA sequencing.docx](https://helsinkifi-my.sharepoint.com/:w:/g/personal/koirala_ad_helsinki_fi/ESi9FDZIT1FEngWSEDwoSqQB8bneTpoJ7qwG-8elG_Goqg?e=YK6CRI) |
| Raw and processed RNASeq data | [RNA seq data_21032023](https://helsinkifi-my.sharepoint.com/:f:/g/personal/koirala_ad_helsinki_fi/ElUVi0H5GpJDmDW1OD4wpggB-Jr_Sussp3wQd9kF9risMA?e=ZbxMv5) |

**Table S2. Target genes and primers used in RT-qPCR**

| Primer name | sequence 5'→ 3' | Target |
| --- | --- | --- |
| Galmu-F | TGCTCGACTATGCCGGTATT | Galactose mutarotases  *L.* *pseudomesenteroides* DSM 20193 |
| Galmu-R | ACCAACCATTACCACCACCA |  |
| Larai-F | GCCTTCATCAATGCACGTCT | L-arabinose isomerase *L.* *pseudomesenteroides* DSM 20193 |
| Larai-R | TCTACCGTCCAACCAAGCTT |  |
| Xyli-F | ACGCTGCTGGACAAATCAAG | Xylose isomerase *L.* *pseudomesenteroides* DSM 20193 |
| Xyli-R | AGAATTGCCCTGTGTAGCCA |  |
| Malpho-F | CGGTGAAAAGCATGGTGTGA | Maltose phosphorylase *L.* *pseudomesenteroides* DSM 20193 |
| Malpho-R | GGCAAGATCCGCAGTTAGTG |  |
| Sucpho-F | TACGTTGGTTTGTTGGCAGG | sucrose phosphorylase *L.* *pseudomesenteroides* DSM 20193 |
| Sucpho-R | AGCCAGCCAAATCAAATGCA |  |
| FruKi-F | CTGGTCATTGCCCATTCCAC | Fructokinase *L.* *pseudomesenteroides* DSM 20193 |
| FruKi-R | CACACCACCACCAAACACAA |  |
| 6pbg1-F | CCCCAATCATCACGGCATTT | 6-phospho-beta-glucosidase *L.* *pseudomesenteroides* DSM 20193 |
| 6pbg1-R | GTGGTTGAGTGTGATGACGG |  |
| F6PPK2-F | TCATTCCGAGCTCACCAAGT | Fructose-6-phosphate phosphoketolase *L.* *pseudomesenteroides* DSM 20193 |
| F6PPK2-R | CAAAGTCAAGTCTTGCGCCT |  |
| Glu6pi-F | CACCAAAGCATCTCAAGGGG | Glucose-6-phosphate_isomerase *L.* *pseudomesenteroides* DSM 20193 |
| Glu6pi-R | ACGGAATGCTTTTGTCGCTT |  |
| LeuDSR4-F | GATGCAGCGATTGAGACAGG | Dextransucrase (dsrD4) *L.* *pseudomesenteroides* DSM 20193 |
| LeuDSR4-R | CCGTTGGCTGCCGTTAAATA |  |
| recA-F | TGAGGTTTACGGGCCAGAAT | Recombinase protein A *L.* *pseudomesenteroides* DSM 20193 |
| recA-R | ACCGCTCCAGATTGTACCAA |  |

**Table S3: qPCR run protocol**

| Heading | Temperature | Time | Cycles |
| --- | --- | --- | --- |
| Initial denaturation | 95 | 3 min |  |
| Denaturation | 95 | 10 sec | 40 |
| Amplification | annealing temp. | 10 sec |  |
| Extension (+Detection) | 72 | 10 sec |  |
| Melting curve↓ |  |  |  |
| Denaturation | 95 | 2 sec |  |
| Melt curve start | 58 | 30 sec |  |
| Increment (+Detection) | 58 to 97 | over 5 min |  |
